# Regulatory T cells in solid organ transplantation

**DOI:** 10.1002/cti2.1099

**Published:** 2020-02-23

**Authors:** Muhammad Atif, Filomena Conti, Guy Gorochov, Ye Htun Oo, Makoto Miyara

**Affiliations:** ^1^ Sorbonne Université Inserm U1135 Centre d'Immunologie et des Maladies Infectieuses (CIMI‐Paris) Hôpital Pitié‐Salpêtrière AP‐HP Paris France; ^2^ Unité de Transplantation Hépatique Hôpital Pitié‐Salpêtrière AP‐HP Paris France; ^3^ Centre for Liver and Gastro Research NIHR Birmingham Biomedical Research Centre University of Birmingham Birmingham UK; ^4^ Academic Department of Surgery University of Birmingham Birmingham UK; ^5^ Liver Transplant and HPB Unit Queen Elizabeth Hospital University Hospital Birmingham NHS Foundation Trust Birmingham UK

**Keywords:** clinical trial, FOXP3, regulatory T cells, safety, transplant, Treg

## Abstract

The induction of graft tolerance remains the holy grail of transplantation. This is important as chronic allograft dysfunction and the side effects of immunosuppression regimens place a major burden on the lives of transplant patients and their healthcare systems. This has mandated the need to understand the immunobiology of graft rejection and identify novel therapeutics. Regulatory T (Treg) cells play an important role in modulating pro‐inflammatory microenvironments and maintaining tissue homeostasis. However, there are fundamental unanswered questions regarding Treg cell immunobiology. These cells are a heterogeneous entity with functionally diverse roles. Moreover, the adoption of novel deeper immunophenotyping and genomic sequencing technologies has identified this phenotype and function to be more complex than expected. Hence, a comprehensive understanding of Treg cell heterogeneity is needed to safely and effectively exploit their therapeutic potential. From a clinical perspective, the recent decade has seen different clinical teams commence and complete first‐in‐man clinical trials utilising Treg cells as an adoptive cellular therapy. In this review, we discuss these trials from a translational perspective with an important focus on safety. Finally, we identify crucial knowledge gaps for future study.

## Graphical abstract

Treg cell therapy has the potential to revolutionise current post‐transplant immunosuppression protocols. Treg cells are presently undergoing early phase trials in solid organ transplantation. However, there are multiple questions regarding Treg cell immunobiology that need to be resolved.

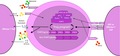

## Introduction

The achievement of graft tolerance remains the holy grail of transplantation. This is clinically driven by the need to prevent chronic allograft dysfunction and minimise the long‐term side effects of immunosuppression.^1^ In this regard, the increased presence of regulatory T cells (Treg cells) within the peripheral circulation and graft microenvironment has been identified as being important in inducing graft tolerance.^2^, ^3^, ^4^ These cells are a part of the adaptive immune system and are considered to have key roles in immunosuppression and homeostasis in different disease settings.^5^, ^6^

**Figure 1:**
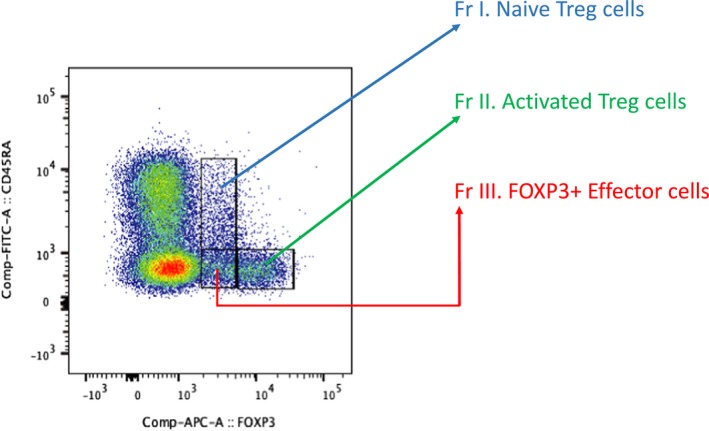
How CD4^+ ^T cells can be split based on FOXP3 and CD45RA expression levels to identify Treg cell subpopulations. The naïve Treg cells are FOXP3^+ ^and CD45RA^+^. However, the activated Treg cells are relatively much more positive for FOXP3^+ ^but CD45RA^−^ instead. Finally, there is an effector T‐cell subpopulation which is also FOXP3^+ ^and CD45RA^−^. This final subpopulation does not have immunosuppressive functions and releases pro‐inflammatory cytokines.

Regulatory T cells in the peripheral circulation are characterised as CD4^+^ with high levels of IL‐2 receptor alpha chain (CD25^high^) and low levels of CD127 and intracellularly, expressing the *forkhead box P3* transcription factor (FOXP3^+^).^5^, ^6^ However, the adoption of novel deeper immunophenotyping technologies has identified this phenotype to be more heterogeneous than initially considered.^6^, ^7^, ^8^, ^9^ (Figure 1) These data differ depending on the species, type of Treg cells, differentiation state and microenvironment.^6^, ^10^, ^11^, ^12^ Hence, a comprehensive understanding of Treg cell heterogeneity is needed to safely and effectively exploit their therapeutic potential. As such, we consider it timely in this review to outline established and novel data regarding Treg heterogeneity and discuss future lines of inquiry.

In solid organ and bone marrow transplantation (SOT and BMT, respectively), Treg cells have been identified as modulators of both T‐cell‐mediated and antibody‐mediated rejection.^13^, ^14^ However, our understanding of the underlying mechanisms is complicated as effector T cells (Teffs) can adopt the Treg‐like phenotype and functions. In reverse, Treg cells can alter their phenotype and functions to adopt a Th17‐like effector cell profile too. It is important to understand these alterations as they can impact the regulatory balance in the graft.^15^ A further limitation is that much of our understanding to date originates from *in vitro* experiments and *in vivo* murine (or non‐human primate; NHP or swine) models.^16^, ^17^, ^18^ It is only in recent years through clinical trials can the *in vivo* relevance of these mechanisms to humans undergoing SOT be deciphered. These trials mainly involve *ex vivo* expansion of autologous Treg cells under Good Manufacturing Practice (GMP) conditions utilising various pharmacological agents that promote their differentiation, expansion, stability and function.^19^ Considering this recent progress, we consider it timely to outline the recent clinical trials in SOT with a focus on safety.

## Heterogeneity of Treg cells

### Treg classification

Polyclonal murine and human Treg cells have been classically classified into three groups: thymic Treg (tTreg), peripheral Treg (pTreg) and induced Treg (iTreg) cells.^10^, ^12^, ^20^ Several authors *controversially* differentiate between tTreg cells and pTreg cells by the higher expression levels of Helios and Neuropilin‐1 (Nrp‐1) on tTreg cells.^12^ Helios is a redundant transcription factor part of the Ikaros family in Treg cells whereas Nrp‐1 is a receptor for class III semaphorins, modulates Treg interactions with dendritic cells,^10^, ^21^ attenuates inflammatory colitis and promotes antitumor immunity.^12^, ^22^ However, Helios/Nrp‐1 on their own cannot categorise tTregs and pTreg cells in humans.^23^, ^24^

A further way of identifying Treg cells is by classifying all CD4^+^ T cells on the basis of CD45RA and FOXP3 expression into three phenotypically and functionally distinct subpopulations.^6^ (Figure 1) These subpopulations include naïve/resting Treg cells (CD45RA^+^FOXP3^+^), activated/effector Treg cells (CD45RA^−^FOXP3^+++^) and FOXP3^+^ effector non‐Treg cells (CD45RA^−^FOXP3^+^). The activated/effector Treg cells are more proliferative and functional as evidenced by higher expression of Ki67 and CTLA4, respectively.

The FOXP3^+^ non‐Treg effector cells are not immunosuppressive and produce cytokines such as IL‐2, interferon‐gamma (IFN‐γ) and IL‐17. The functional role of FOXP3 in the naïve and activated Treg cells is further reinforced by the finding that their FOXP3 regions are mostly demethylated in comparison with that of the non‐suppressive FOXP3^+^ effector cells. However, both FOXP3 and the Treg‐specific demethylated region (TSDR) are intracellular entities so challenging to adapt to cellular therapy applications in patients. Hence, identification of novel cell‐surface markers such as sialyl Lewis x (CD15s) whose expression is strongly correlated with a highly suppressive effector Treg is necessary.^25^, ^26^ This can also facilitate the exclusion of non‐suppressor FOXP3^+^ cells, which have the potential for effector function.^6^

**Figure 2:**
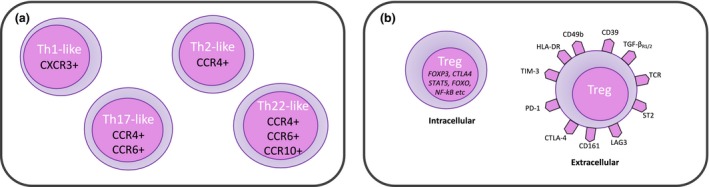
Diversity of Treg cells and their subpopulations. **(a)** shows Treg cells split into distinct populations depending on their expression profile of chemokine receptors. **(b)** shows a range of intracellular and/or extracellular markers identified on Treg cells. These markers are not exhaustively demonstrated in the diagram but are to give an indicator of the complexity of whichever phenotypic classification one utilises.

A further subdivision has been through identifying chemokine receptors to split effector Treg cells (CD25^hi^CD127^lo^CD45RO^+^) into co‐existing T‐helper (Th)‐like Treg cells: Th1 (CXCR3^+^), Th2 (CCR4^+^), Th17 (CCR4^+^CCR6^+^) and Th22 (CCR4^+^CCR6^+^CCR10^+^) Treg cells^9^ (Figure 2). This was matched by their cytokine release profile as, although both Th1‐like and Th17‐like Treg cells produced IL‐10, each subset, respectively, produced IFN‐γ and IL‐17 only. Interestingly, their Th2‐like and Th22‐like counterparts did not produce any IL‐10 or IL‐4 or IL‐22, and the lack of the latter two would be consistent with the anti‐inflammatory function of Treg cells. These findings are important for two key reasons: (1) homing potential and (2) like‐for‐like interaction. In terms of homing potential, the ability to isolate, modulate and infuse Treg cells with specific chemokine markers would optimise their ability to home towards specific sites of alloreactive cell contact (e.g. allograft, lymph nodes).^27^, ^28^ In terms of like‐for‐like interaction, the selection of similar Th‐subtype Treg cells could optimise their potential to modulate similar Th‐subtype effector responses.^28^, ^29^

In parallel with the above classifications, other authors have adopted similar approaches to investigate Treg cell subpopulations using markers such as CD161, CD45RO, CD39, CD49d, programmed death‐1, T‐cell immunoreceptor with Ig and ITIM domain (TIGIT), and T‐cell immunoglobulin and mucin domain 3 (TIM‐3) to elucidate subpopulation‐level heterogeneity.^9^, ^30^, ^31^, ^32^, ^33^, ^34^ (Figure 2) Establishing mechanistically relevant links between these subpopulations, their markers and FOXP3 (or other transcription factors) is further complicated by the fact that non‐FOXP3‐expressing T cells can be induced or genetically modified to express FOXP3 and adopt Treg‐like functions too.^35^, ^36^, ^37^, ^38^ This is important clinically as increased FOXP3 transcripts and Treg cells have been identified in patients developing operational tolerance or reduced rejection after SOT.^39^, ^40^ Putting all this together, there is a need to delineate Treg heterogeneity using novel immunophenotyping approaches.

### Understanding Treg biology via novel technologies

The adoption of novel technologies such as cytometry by time of flight and single‐cell RNA sequencing (scRNAseq) has uncovered further Treg heterogeneity.^7^, ^8^, ^41^, ^42^, ^43^ In one study investigating Treg cells (CD4^+^CD25^+^CD127^lo^) from peripheral blood, 22 novel subpopulations were identified using a range of markers such as HLA‐DR, CD62L, CD27 and ICOS (in addition to the markers discussed above).^41^ This has been studied further by other teams in a range of transplant‐related (renal/liver) and non‐transplant‐related settings, respectively.^7^, ^42^, ^43^, ^44^ In the case of liver transplantation, a 22‐marker panel identified the presence of significantly more Treg cells (CD4^+^CD25^+^FOXP3^+^) in their ‘tolerant’ cohort post‐transplant compared to controls on immunosuppression.^42^ However, it was a novel *non‐Treg* T‐cell subset (CD4^+^CD25^+^CD5^+^CD38^−/lo^CD45RA^+^) that correlated specifically with tolerance in these children post‐transplant.

Using a different technology, Zemmour *et al*.^8^ performed scRNAseq on human and murine Treg and Teffs and demonstrated distinct transcriptomic profiles between both cell types. In this study, human Treg and effector cells were sorted as CD4^+^CD25^+^CD127^lo^ and CD4^+^CD25^−^CD127^hi^, respectively, whereas their murine counterparts from FOXP3^gfp^ mice were sorted as CD4^+^TCRβ^+^GFP^+^ or CD4^+^TCRβ^+^GFP^−^ instead. With their murine cells, they could reliably differentiate between Treg cells and Teffs by identifying transcripts specific to both – such as their origin (Helios), function (FOXP3, IL‐2, CTLA4, OX40, GITR) and others which may be related to metabolic processes but require further study (e.g. folate receptor, GPR43 and a mitochondrial crista protein). Importantly, all the genes for these transcripts are targets of FOXP3 transcription factor, thus further demonstrating its critical role in Treg function.

However, in humans, although both cells were identified in distinct clusters using dimensionality reduction analysis, up to 55% of human Treg cells were found overlapping with Teffs. These overlapping Treg cells (described as ‘furtive’ Treg cells by the study authors) had lower levels of FOXP3 expression than *bona fide* Treg cells in humans. Whilst the mechanism for this was not delineated, this indicates that the link between FOXP3 expression and the CD4^+^CD25^+^CD127^lo^ phenotype is not as direct as previously described.^45^ This is important from a clinical perspective as current in‐human trial protocols are expanding Treg cells with differing phenotypes: CD4^+^CD25^+^ (e.g. TRACT trial) or CD4^+^CD25^+^CD127^lo^ expression (e.g. LITTMUS/TASK trials). Although FOXP3 expression may form part of the product release criteria, it is uncertain as to how stable FOXP3 expression will be *in vivo* and which other transcription factors may be necessary to maintain *in vivo* Treg function.

### How important is FOXP3 expression in Treg cells?

**Figure 3:**
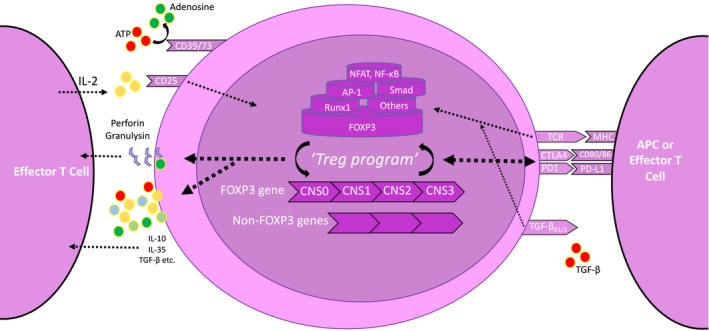
Schematic demonstration of a Treg cell and the contributors to the Treg program. Treg cells can be modulated by numerous mechanisms; T‐cell receptor (TCR) stimulation from antigen‐presenting cells (APCs) or effector T cells; cytotoxic T‐lymphocyte antigen 4 (CTLA4); programmed death 1 (PD1); and interleukin‐2 (IL‐2) via CD25. There is a complex interaction between FOXP3 and other transcription factors [e.g. *nuclear factor of activated T cells* (NFAT), *activator protein‐1* (AP‐1)], and these interact with the FOXP3 gene across the different loci. Although several intracellular mechanisms are triggered, they all centre on the crucial cross‐talk between the FOXP3 gene and others to transcribe an optimal ‘Treg program’. This ‘Treg program’ is then put into action via protein translation and ultimately facilitates Treg function via the numerous mechanisms illustrated.

In spite of the adoption of novel approaches, the common theme through all immunomonitoring studies is the use of FOXP3 as a reliable signature of Treg lineage.^10^, ^20^, ^35^ This is a consistent finding based on data demonstrating that either genetic or pharmacological induction or inhibition of FOXP3 (in humans and mice) led to critical alterations in the function of the manipulated cell – no matter whether this was initially a *bona fide* Treg or an Teff.^35^ However, although the Treg signature is defined mostly in the thymus,^46^ this begs the questions: what contributes to the remainder of the tTreg transcriptomic signature and how are both tTreg transcriptomic signature and function maintained in the graft/lymphoidal microenvironment? (Figure 3) This is important as FOXP3 expression alone is insufficient to maintain a stable Treg transcriptomic signature and function.^47^

FOXP3 interacts with numerous transcription factors such as *nuclear factor of activated T cells*, *activator protein‐1* and forkhead box amongst others.^48^, ^49^, ^50^ These complexes bind to the *conserved noncoding sequences* (CNS1–3) on the FOXP3 locus to promote particular transcripts such as *il2ra* and *ctla4* indicative of the ‘Treg program’^35^, ^47^, ^51^ (Figure 3). This locus of interest has been further expanded because of the discovery of the CNS0 region and recent work on super‐enhancers.^52^ This was mainly based on Satb1 (chromatin‐organising protein), whose deficiency was linked to suboptimal Treg‐specific super‐enhancer activation and thereby negatively altered the initial development of tTregs.^52^

Taking all these data into account, the focus on FOXP3 is likely rather reductionist as experiments involving transfection of FOXP3 in naïve T cells have demonstrated that only a portion of the *bona fide* Treg signature was induced (e.g. *ctla4, il2ra, nrp1, tnfrsf18*).^46^, ^53^ Some of these genes are FOXP3‐dependent and some not.^53^, ^54^ They can also be influenced by TCR‐, IL‐2‐ and TGF‐β‐based signalling cascades.^46^ (Figure 3) These data indicate that the overall Treg signature is a complex interplay between FOXP3 coregulatory genes, regulators upstream of FOXP3 and parallel pathways (TCR, IL‐2, TGF‐β).^46^ This may explain why FOXP3^−^ Treg cells continue expressing mainly Treg‐like transcriptional signatures in spite of the lack of FOXP3 as there could be underlying additional mechanisms either intrinsic to Treg cells or the microenvironment. From a transplant perspective, it is important to understand how these regulatory elements can mechanistically influence Treg cell and effector T‐cell function in the graft/lymphoidal tissue.

One of the mechanisms in promoting FOXP3 expression may involve epigenetic regulation.^51^, ^55^ Stable FOXP3 expression is dependent on FOXP3 protein and histones acetylation and the ability of FOXP3 and its coregulatory transcription factors to bind to the TSDR – found on CNS2 of the FOXP3 locus.^51^, ^56^ (Figure 3) This region is partially demethylated in iTreg cells (thus potentially explaining their instability) and fully methylated in Teffs.^10^, ^12^, ^20^ However, although the continual demethylation status may be necessary to maintain FOXP3 expression, the function of Treg cells is in parallel also dependent on CpG demethylated patterns on other genes such as *il2ra*, *ikzf4* and *ctla4* amongst others.^47^ These patterns are optimally modulated in the periphery, which suggests that recently emigrated tTreg cells still need to maturate – again because of a pre‐set Treg‐intrinsic program or tissue‐specific immunoregulation. Unsurprisingly, the loss of demethylated patterns and FOXP3 expression negatively impacts on Treg function.^57^

Considering the role of FOXP3, its stability and expression is a key aspect during the manufacturing of the Treg cell product. FOXP3 expression can be maintained by utilising DNA methyltransferase inhibitors (DNMT; azacytidine) and histone deacetylase inhibitors (HDAC; voronistat) during *ex vivo* Treg expansion.^26^ DNMT inhibition aims to maintain the demethylated patterns as discussed above whereas HDAC inhibition aims to maintain the acetylation status of histones and perhaps of FOXP3 and thereby optimise its expression.^26^, ^58^ Using both DNMT and HDAC inhibitors (azacytidine and voronistat), Treg function was enhanced *in vitro* and *in vivo* within a xeno–graft‐versus‐host‐disease (xeno‐GvHD) model.^26^ Azacytidine targets the 10–11 translocation enzyme (TET) family (specifically TET2/3) to optimise the stability of FOXP3 expression.^59^, ^60^ The importance of this approach was also illustrated by Tao *et al*.*,* 58 who induced tolerance to cardiac and islet allografts in murine models through administering a combination of rapamycin and a pan‐HDAC inhibitor (trichostatin‐A). However, these data involved pan‐inhibition of their relevant enzymes and so did not reflect the effects of modulating their numerous isoforms. This is important as divergence in the inhibition of different isoforms and their effects on Treg function is known.^61^, ^62^ The authors also used an HDAC9 KO murine model to demonstrate a boost to the quantity of Treg cells and their function. Similar effects have also been demonstrated in the studies focussing on inhibition of HDAC3/10/11 either pharmacologically or via enzyme KO or Treg‐specific enzyme KO.^63^, ^64^, ^65^ Putting this together, although these studies aim to elicit the effects of targeted enzyme isoform modulation, it is just as important to delineate the mechanisms underlying interactions between these isoforms and FOXP3 (or other transcription factors) and how this influences Treg phenotype/function.^64^ The specificity of any epigenetic modulation is important to ensure that only the intended effect on Treg phenotype/function is achieved and any off‐target effect is minimised.

## Rationale for clinical trials

In spite of the pre‐clinical interest in delineating the cross‐talk between FOXP3 and Treg cell immunosuppressive function, it is possible to incorporate FOXP3 measurements as part of GMP‐expansion protocols and use these as surrogate indicators of Treg cell product quality and potency.^66^, ^67^ This is important as Treg cell therapy in transplantation could more accurately target the anti‐rejection response whilst having minimal off‐target effects and chronic allograft toxicity. This is of major interest to our patients who currently adhere to differing long‐term immunosuppression regimens to prevent graft rejection. These decisions regarding immunosuppression have been made in consideration of the harmful consequences of long‐term drug toxicity as well as aiming to maintain a functional immune system against pathogens/tumors. In addition, our transplant patients are often multi‐morbid so it is important to ensure that any transplant‐related pharmacotherapy does not interfere with other drugs or impact on their quality of life.^68^ Hence, the successful translation of Treg cell therapy could benefit our patients in many ways.

## Clinical trials: today and tomorrow

The recent decade has seen the delivery of first‐in‐man clinical trials of Treg cellular therapies for a range of transplant‐related and non‐transplant‐related indications.^66^, ^69^, ^70^ Although these trials have focussed on safety, a core theme across all is the heterogeneity in GMP protocols with regard to cell isolation, manipulation, expansion, dosing, specificity and post‐administration cell tracking. Whilst some consensus towards standardisation was developed in the multi‐centre ONE Study consortium^71^ (Treg cells cellular therapy in kidney transplantation), the reality is that the novelty of Treg cells mandates numerous in‐human studies focussing on optimisation and standardisation of GMP conditions. In Table 1, we outline these current trials involving Treg cells cellular therapy in SOT and BMT.

**Table 1:** Registered trials on Clinicaltrials.gov as of 28 April 2019 involving regulatory T‐cell therapy in transplantation

Organ/tissue transplanted	Trial ID/Name	Lead institution(s)	Dosing	Nature of cell product
Liver	Todo/Okumura^70^	Hokkaido, Japan	8.92–37.7 × 10^6^/kg cells but 0.23–6.37 × 10^6^/kg Treg cells	Donor‐specific and with costimulation blockade
Liver (2–6 years post‐transplant)	ARTEMIS (NCT02474199)	UCSF, USA	300–500 × 10^6^ cells	Donor‐specific
Liver (12 weeks post‐transplant)	dELTA (NCT02188719)	UCSF, USA	25–960 × 10^6^ cells	Donor‐specific
Liver	LITTMUS‐UCSF (NCT03654040)	UCSF, USA	Target: 100–500 × 10^6^ cells	Donor‐specific
Liver	LITTMUS‐MGH (NCT03577431)	MGH, USA	Target: 2.5–500 × 10^6^ cells	Donor‐specific and with costimulation blockade
Liver	ThRIL (NCT02166177)	King's College Hospital, UK	0.5–6.5 × 10^6^/kg	Polyclonal
Liver	NCT01624077 (First Trial)	Nanjing, China	1 × 10^6^/kg at several intervals	Polyclonal
Liver	NCT01624077 (Second Trial)	Nanjing, China	1 × 10^6^/kg at several intervals	Donor‐specific (MHC peptides)
Kidney	TRACT^66^	Northwestern, USA	500–5000 × 10^6^ cells	Polyclonal
Kidney	TASK *preliminary* 69	UCSF, USA	Target: 320 × 10^6^ cells	Polyclonal, deuterated
Kidney	TASK (NCT02711826)	UCSF, USA	300–500 × 10^6^ cells	Polyclonal vs donor‐specific
Kidney	NCT03284242	Kentucky, USA	Unknown	Unknown
Kidney	ONE Study NCT02091232	MGH, USA	Unknown	Donor‐specific and with costimulation blockade
Kidney	ONE Study NCT02244801	UCSF, USA	300–900 × 10^6^ cells	Donor‐specific
Kidney	ONE Study NCT02371434	Charité, Germany	0.5–3 × 10^6^ cells kg^−1^	Polyclonal, fresh
Kidney	ONE Study NCT02129881	Kings College London, UK	1–10 × 10^6^ cells	Polyclonal, frozen
Kidney	NCT03867617	University Hospital Regensburg, Germany, and Medical University of Vienna, Austria	Unknown	Clonality unknown. Protocol involves donor BMT too
Kidney	NCT01446484	Pirogov, Russia	2 doses of 200 × 10^6^ cells	Polyclonal
Islet	NCT03444064	Alberta, Canada	400–1600 × 10^6^ cells	Polyclonal

### Liver

The first published trial using these cells in SOT was by Todo *et al*.^70^ from Hokkaido (Japan), who generated a donor‐specific cell product with a view to tolerance induction after liver transplantation. Ten patients with end‐stage liver disease received a graft from a living donor. The recipients also underwent a splenectomy on the day of transplant and were subsequently on 1 month of mycophenolate (MMF) and long‐term tacrolimus/cyclosporine. They were weaned off from 6 months onwards if graft function was stable and all immunosuppression was withdrawn at 18 months. Before the transplant, the team collected 4–5 × 10^9^ cells each from the donor and recipient. The recipient and irradiated donor cells were co‐cultured with antagonist anti‐CD80/86 antibodies for 2 weeks. After 2 weeks, the remaining cells had to be more than 80% viable with > 1 × 10^6^/kg being CD4^+^CD25^+^FOXP3^+^ to be eligible for recipient administration on post‐operative day 13. The final doses administered were 8.92–37.7 × 10^6^/kg cells of which 0.23–6.37 × 10^6^/kg were Treg cells. In terms of Treg percentage, this would equate to a range of between 2.6% and 16.9% Treg cells out of all infused cells. Importantly, there was no reported significant adverse event (SAE) and seven patients were weaned off immunosuppression by 18 months.

It is important to contextualise this weaning period with regard to other published data. In this study, weaning started at 6 months post‐transplant and, thus, took place over the next 12 months. In another adult cohort undergoing a liver transplant, 77 patients underwent immunosuppression weaning over 15 months.^72^ Ten of them successfully remained off immunosuppression for 1 year without *clinical evidence of* rejection and nine continued as such for 2 years altogether. In comparison, in another adult cohort, 102 patients who were already at least 3 years post‐liver transplant were weaned off immunosuppression over 6–9 months.^73^ The 33 patients who successfully remained off immunosuppression for 1 year after withdrawal had been on immunosuppressants for longer, avoided receiving calcineurin inhibitors and were older. A further study involving 20 paediatric liver transplant recipients was able to successfully withdraw immunosuppression in 12 patients over a period of at least 9 months.^74^ These 12 patients remained off immunosuppression for on average 3 years (median 35.7 months). All these data indicate that the weaning period employed by Todo *et al*.^70^ is not in an unfamiliar range to that employed by other studies. Most importantly, we must emphasise that there is a lack of a robust evidence base regarding optimum weaning times for patients on immunosuppression regimens. This is being complicated further by Treg‐based cellular therapies.

Five patients demonstrated *in vitro* donor‐specific nonresponsiveness and two were hyporesponsive, thus implying a degree of donor‐antigen specificity of their co‐cultured cell product. However, from a mechanistic perspective, not only did the co‐cultured cells inhibit donor‐specific responses, but at more concentrated doses, third‐party tolerogenic responses were also elicited during mixed lymphocyte reaction experiments. It is also important to note that the specific role of Treg cells in mediating the reported hypo‐ or nonresponsiveness cannot be concluded as patients received an infusion containing all cultured cells rather than Treg cells only. Hence, the possibility of other mechanisms such as indirect Treg‐based immunoregulation (e.g. via regulatory dendritic cells or macrophages) or multi‐cellular immunoregulation (e.g. anergy induction, infectious tolerance, bystander suppression) cannot be excluded.^75^, ^76^, ^77^ Nevertheless, these data suggest this co‐cultured cell product was more potent towards donor‐specific antigens than third‐party cells. The underlying mechanisms of operational tolerance induced by this cell therapy are yet undiscovered.

It is noteworthy that the three patients who developed acute cellular rejection during the immunosuppression weaning process all underwent transplantation for autoimmune liver disease. This could imply that the immunoregulatory threshold for these patients is much higher, and thus, achieving long‐term control of effector responses could be more challenging.

Overall, this study has demonstrated the feasibility of their approach in producing a potent anti‐donor cellular product with the potential for tolerance induction. The long‐term outcomes of this study are awaited.

#### Trials in progress

In contrast to the study above, other transplant centres have focussed on manufacturing a ‘purer’ Treg cell product with/without alloantigen specificity instead (See Table 1). This line of research is based on data demonstrating that donor‐reactive Treg cells better suppress the alloreactive effector T‐cell response than polyclonal Treg cells.^78^ It is important to elucidate and exploit this mechanism in humans as the extent of product purity and alloantigen specificity could have implications for therapeutic potency and infused cell numbers.^79^, ^80^, ^81^ The teams from Nanjing (China) and Kings College Hospital (United Kingdom) are focussing on polyclonal Treg cells in their trials (NCT01624077 and NCT02166177). In comparison, one arm of the LITTMUS trial (NCT03577431, NCT03654040) at Massachusetts General Hospital (MGH) will infuse alloantigen‐specific Treg cells (CD4^+^CD25^+^CD127^lo^) with exposure to costimulatory blockade at doses of 2.5–500 × 10^6^ cells. Whereas, the second arm of the LITTMUS trial at University of California, San Francisco (UCSF), will infuse alloantigen‐specific Treg cells (CD4^+^CD25^+^CD127^lo^) without exposure to costimulatory blockade at doses of 100–500 × 10^6^ cells instead. In comparison, two other trials from UCSF (ARTEMIS and dELTA trials) are also producing donor alloantigen‐specific Treg cells; however, their Treg cells will be administered in patients who are 2–6 years or 12 weeks post‐transplant, respectively. By comparing these latter two trials, one could generate critical hypotheses regarding the optimal time points for Treg infusion. All these data collectively would also improve our current understanding of post‐transplant immunoregulatory and tolerance induction mechanisms.

### Kidney

In parallel, similarly, novel work has also been undertaken by multiple centres globally in kidney transplantation. A key advantage of the kidney is that there is a larger scope logistically for living donors, which provides continuous access to fresh donor antigens and, thus, donor‐specific Treg cells.^66^

#### TRACT trial

The TRACT trial from Northwestern University (Chicago, USA) utilised *ex vivo* expanded polyclonal Treg cells infused in a dose‐escalation manner (500–5000 × 10^6^ cells) into nine patients who had received renal allografts from living donors.^66^ Patients received alemtuzumab on the day of transplant and post‐operative day 2 and were maintained on MMF and tacrolimus (switched to sirolimus on day 30). The Treg cells were subsequently infused on day 60. There were no cases of opportunistic infections resulting from cytomegalovirus or polyoma virus nor any cases of rejection for the 2 years reported.

Their Treg cells were isolated using the CliniMACS system and expanded *ex vivo* for 3 weeks using agonist anti‐CD3/anti‐CD28 beads, IL‐2, transforming growth factor‐beta (TGF‐β) and sirolimus. The product purity was > 98% CD4^+^CD25^+^ and > 80% FOXP3^+^ with similarly high demethylation status (although not 100%). These latter facts are important as demethylation is crucial for Treg cell stability, and an incompletely demethylated FOXP3 gene can lead to the conversion of Treg cells into effector cells.^10^, ^35^, ^46^, ^51^ However, as the demethylation status of Treg cells in this trial was high, one would expect their efficiency close to those of *bona fide* Treg cells. This is based on our discussion earlier regarding Treg subpopulations of which the naive Fr I and activated Fr II subpopulations have been shown to be 85–100% demethylated.^6^

Other mechanistic experiments in this trial also focussed on the expression of Treg cells markers of function (CTLA4, CD62L), homing (CXCR4) and differentiation status (CD45RO). All functional markers were significantly increased post‐expansion, and more than 80% of CD4^+^ cells were CD45RO^+^, thus indicating their Treg cells were matured at the end of the expansion period. All these data are further enhanced by the data from *in vitro* suppression assays confirming superior Treg suppressive capacity at the end of the expansion period. Putting all these together, this trial reported no cases of opportunistic infections or rejection after the infusion of polyclonal Treg cells in patients undergoing *de novo* kidney transplantation. The next step will be to demonstrate the *in vivo* survival and function of these Treg cells.

#### TASK trial

In comparison, the preliminary TASK trial from UCSF focussed on utilising polyclonal Treg cells to modulate subclinical inflammation in the renal allograft.^69^ This is an important study question as subclinical inflammation because of its chronic low‐grade nature is not promptly diagnosed and can facilitate delayed allograft dysfunction. The authors recruited three patients who demonstrated such inflammation (Banff i‐ and t‐ < 2)^82^ on their 6 months post‐transplant biopsy. Patients were already on MMF, tacrolimus and prednisolone when they had their Treg cells acquired. Importantly, from a safety perspective, the authors reported no SAEs as a direct result of their Treg product and there were no reported infections or graft dysfunction during the 1‐year follow‐up period. The patients received *ex vivo* expanded autologous Treg cells at an average dose of 320 × 10^6^ cells. These cells were isolated as CD4^+^CD25^+^CD127^lo^ via flow‐activated cell sorting (FACS). They were expanded for 14 days using anti‐CD3/anti‐CD28 activation beads, IL‐2 and deuterated glucose. Their expanded Treg cells were > 97% CD4^+^ and > 93% FOXP3^+^ with extremely low percentage (< 0.37%) of contaminating CD8^+^ cells. It is already clear that there are numerous differences between this trial and the TRACT trial above in regard to pathology, Treg dosing, isolation and expansion protocols.^66^, ^69^ A novel development in this trial was the use of deuterated glucose to facilitate *in vivo* infused Treg tracking. This approach was previously used by the group in a Treg cell therapy trial in type 1 diabetes.^83^ However, a critical limitation from the current trial was that the deuterated signal was undetectable by 3 months – compared to being detectable over 12 months in their previous trial. This negates the long‐term use of this technique. There were no reported data identifying these Treg cells at the 2 weeks and 6 months post‐infusion renal biopsies. Therefore, it is unclear whether their deuterated Treg cells' product was able to migrate into the graft. This is supplemented by the fact that no patient demonstrated an increase in FOXP3^+^ % expression in the renal biopsy at 2 weeks post‐infusion and only 1 did so at 6 months. Moreover, although this technique facilitates short‐term tracking, it does not provide knowledge regarding *in vivo* Treg function. All in all, no SAEs occurred after the infusion of FACS‐isolated and polyclonally expanded deuterated Treg cells in the context of kidney transplantation. This work is being built upon currently through the TASK trial (NCT02711826), which will compare the efficacy of polyclonal and alloantigen‐specific Treg cells.

#### Trials in progress

The ONE Study consortium aimed to assess the feasibility and safety of novel cellular therapies in kidney transplantation.^84^ A key aspect of this consortium was that different centres adopted a common immunosuppression protocol (tacrolimus, mycophenolate and 3 months of steroids) with the critical difference being the type of cell therapy product utilised. The centres at Charité (Germany), Oxford & Kings College London (UK), UCSF and MGH (USA) focussed on developing different forms of Treg cell therapies (See Table 1). The process of conducting this study also highlighted the novel opportunities and challenges associated with manufacturing and monitoring the use of these different cell therapies. These include aspects such as getting regulatory authority approvals, setting up a GMP facility, patient recruitment, immunomonitoring and analysing large‐scale datasets. Although these trials were not powered to test efficacy, it is noteworthy that the 12 patients treated in the UK with polyclonal Treg cells did not experience acute rejection as per biopsy.^84^ The recent study reports also suggest that patients were able to reduce their immunosuppression and, most importantly, that ‘cell therapy…is safe’.^84^ We await the publication of these datasets. Initial immunomonitoring analysis was presented at the recent American Transplant Congress 2019.^85^ All of these data will be crucial in enhancing our early understanding of Treg‐based cell therapy in clinical kidney transplantation. A positive advance already is that the Oxford group is undertaking the Phase II stage (TWO Study), which will aim to test the efficacy of Treg cells in preventing graft rejection.^86^ The results of this trial and those of other centres are awaited with great interest.^84^

## Long‐term safety issues

Due to the recent nature of Treg cell therapy trials in transplant, only short‐term issues can be initially identified. However, it is the long‐term safety issues classically associated with mainstream immunosuppressive drugs such as opportunistic infections, malignancies and chronic allograft dysfunction that need to be closely monitored for too. In addition, there may also be other Treg‐specific safety issues uncovered of which we are currently unaware. These issues require the implementation of regular clinical follow‐up, immunomonitoring and broader pharmacovigilance (potentially via a cell therapy registry). In the meantime, considering the pre‐experimental data involving Treg cells, there may be particular Treg‐specific safety issues that we can at least pre‐empt.

### Treg cell plasticity

The potential for Treg cells to adopt a Th17‐like effector cell phenotype and function in an inflammatory microenvironment is a critical safety concern. This is because Th17 cells are elevated in patients with acute and chronic allograft rejection and, thus, may contribute towards an exacerbation of both conditions.^87^, ^88^ This is supported by murine data demonstrating Th17 cells as key players in the promotion of allograft rejection.^89^, ^90^ Treg cells when stimulated in the presence of pro‐inflammatory cytokines such as IL‐1, IL‐2, IL‐15, IL‐21 and IL‐23 alongside allogeneic monocytes can upregulate the Th17 lineage transcription factor, retinoic‐acid‐receptor‐related orphan receptor gamma, and secrete IL‐17.^91^ Another pathway involving Treg‐Th17 cross‐talk lies in the fact that both originate from naïve CD4^+^ cells in the first place.^92^ However, their subsequent differentiation pathway is determined by the presence of TGF‐β without IL‐6 (for Tregs) or with IL‐6 (for Th17) in the microenvironment, respectively.^92^

This potential of the microenvironment to convert Treg cells into rejection‐propagating Th17 cells could dictate the time point at which to infuse Treg cells. In theory, Treg cell therapy could be provided proactively (i.e. before patients develop rejection) or reactively (i.e. given to patients with diagnosed acute or chronic rejection). However, it is unknown as to whether Treg cells can induce tolerance in a microenvironment already infiltrated by Teffs. In one murine study, it was shown that Treg cells can prevent the initial activation of only resting Teffs.^93^ However, in a different study involving RAG knockout mice, the adoptive transfer of Treg cells was unable to prevent the onset of autoimmunity in those mice that had previously been administered Teffs.^94^ Moreover, Teffs can be resistant to the direct effects of Treg cells.^94^ If these findings were directly translated into the clinic, it would mean providing Treg cell therapy proactively to prevent the onset of rejection in the first place. However, even then the question would be how to stratify patients.

### Bystander suppression

A potential problem with Treg cell therapy could arise as Treg cells once activated can perform their suppressive function in a non‐antigen‐specific manner.^95^ This effect known as ‘bystander suppression’ was identified in transgenic murine models whereby antigen‐specific CD4^+^CD25^+^ T cells initially required contact with a complementary epitope for TCR‐mediated activation.^95^ However, upon being activated these cells were able to suppress effector CD4^+^ T cells specific for third‐party antigens too. This effect has also been demonstrated in an *in vivo* murine model of allogenic skin transplant.^96^ If indeed this phenomenon is replicated in our patients too, there could be implications with regard to modulating antigen‐specific rejection whilst maintaining immunity towards pathogens and potential tumoral neoepitopes.

## Future perspectives

In the end, the discussions concerning Treg heterogeneity are to aid the development of clinically safe protocols in which Treg cells can be manipulated and manufactured. To this day, two main approaches could facilitate this in humans: *in vivo* expansion of Treg cells and *ex vivo* Treg expansion under GMP conditions.^10^, ^26^, ^67^, ^71^ The former approach has been the subject of clinical trials involving low‐dose IL‐2 in the transplant setting (e.g. NCT02739412, NCT02949492, NCT02417870). However, the majority of centres are focussing on *ex vivo* Treg expansion instead. Comparing data from both these approaches will be critical in identifying the optimal method of exploiting Tregs clinically.

A further critical question going forward will be immunosuppression management in Treg cell therapy trials. Depending on the transplant centre protocol, different patients currently are subject to different immunosuppression regimes (e.g. steroid‐sparing, deferring the introduction of calcineurin inhibitors) with differing doses. It is important to take these immunosuppressive agents into account when designing trials as concurrent immunosuppression can modulate Treg cell survival and function. This aspect has been comprehensively discussed previously by Furukawa *et al*.^97^

Another unaddressed issue is whether genetic modification of Treg cells could augment their *in vivo* modulation of graft rejection. A full discussion of genetic modification has been beyond the scope of this review; however, we reference here some key articles from teams that have developed FOXP3‐overexpressing Tregs, T‐cell‐receptor‐transgenic, TCR‐Tg Treg, and chimeric‐antigen‐receptor‐specific, CAR‐Tregs.^38^, ^79^, ^98^

With regard to Treg heterogeneity, although novel technologies may facilitate deeper comprehension of Treg cells and their subsets, the adoption of these technologies for routine clinical use is impractical and financially unviable. Hence, the key question is, *how deep do we need to go?* It may be that we only utilise novel and expensive technologies for pre‐clinical experiments to identify and validate the Treg program for optimal *in vivo* activity. These data could then be reverse engineered and adapted to clinically available assays to facilitate use in larger multi‐centre trials.

With all these in mind, there are numerous questions regarding cell product composition and transplant protocols (Table 2) that require addressing if the field of Treg cell therapy in SOT is to progress.

**Table 2:** Key *known unknowns* in the field of Treg‐based cell therapy for transplant‐related applications

Known unknowns
Identification and Selection
What are the ideal Treg cell phenotypic identifiers?
What level of specificity (polyclonal, TCR Tg or CAR) is required?
Is there a need to select Treg cells with transplanted graft‐specific homing potential?
Pharmacology
What is the optimal Treg infusion dose?
When should Treg cells be administered in a transplant protocol?
Will one or multiple Treg cell infusions be required to reach the trial end‐point?
How can *in vivo* Treg cell pharmacokinetics and pharmacodynamics be monitored/traced?
Maintaining Treg cell function
What are the ideal tissue sites (lymph nodes, thymus, graft) for immunoregulatory activity?
Which is the optimal immunosuppression regimen to maintain *in vivo* Treg cell function?
What is the role of the microenvironment in maintaining *in vivo* Treg cell function?
What is the optimal Treg epigenetic program to maintain their *in vivo* survival and function?
Pharmacovigilance
What are the long‐term consequences of Treg cell therapies?
Is an international patient registry feasible?

The data we glean from first‐in‐man clinical trials and pre‐clinical studies will help answer the critical *known unknowns* (Table 2).^19^ However, we recognise that the novel nature of Treg cells means that there are numerous other *unknown unknowns* that we will only begin to realise in the coming years. Moreover, it will only be through multi‐centre collaborations that we can address a range of these new aspects of translational Treg cell biology. Indeed, there has never been a more exciting time to be involved in the field of Treg cell therapies in transplantation. The successful translation of Treg cell therapies could transform the lives of our transplant patients.

## Conflict of interests

The authors declare no conflict of interest.

## References

[1] Christians U , Klawitter J , Klawitter J *et al* Biomarkers of immunosuppressant organ toxicity after transplantation: status, concepts and misconceptions. Expert Opin Drug Metab Toxicol. 2011; 7: 175–200. 21241200 10.1517/17425255.2011.544249 PMC3079351

[2] Bahmani B , Uehara M , Jiang L *et al* Targeted delivery of immune therapeutics to lymph nodes prolongs cardiac allograft survival. J Clin Invest 2018; 128: 4770–4786. 30277476 10.1172/JCI120923 PMC6205374

[3] Graca L , Cobbold SP , Waldmann H . Identification of regulatory T cells in tolerated allografts. J Exp Med 2002; 195: 1641–1646. 12070291 10.1084/jem.20012097 PMC2193557

[4] Lee I , Wang L , Wells AD *et al* Recruitment of Foxp3^+^ T regulatory cells mediating allograft tolerance depends on the CCR4 chemokine receptor. J Exp Med 2005; 201: 1037–1044. 15809349 10.1084/jem.20041709 PMC2213137

[5] Mohr A , Atif M , Balderas R *et al* The role of FOXP3^+^ regulatory T cells in human autoimmune and inflammatory diseases. Clin Exp Immunol 2019; 197: 24–35. 30830965 10.1111/cei.13288 PMC6591146

[6] Miyara M , Yoshioka Y , Kitoh A *et al* Functional delineation and differentiation dynamics of human CD4^+^ T cells expressing the FoxP3 transcription factor. Immunity 2009; 30: 899–911. 19464196 10.1016/j.immuni.2009.03.019

[7] Kordasti S , Costantini B , Seidl T *et al* Deep phenotyping of Tregs identifies an immune signature for idiopathic aplastic anemia and predicts response to treatment. Blood 2016; 128: 1193–1205. 27281795 10.1182/blood-2016-03-703702 PMC5009512

[8] Zemmour D , Zilionis R , Kiner E *et al* Single‐cell gene expression reveals a landscape of regulatory T cell phenotypes shaped by the TCR. Nat Immunol 2018; 19: 291–301. 29434354 10.1038/s41590-018-0051-0 PMC6069633

[9] Duhen T , Duhen R , Lanzavecchia A *et al* Functionally distinct subsets of human FOXP3^+^ Treg cells that phenotypically mirror effector Th cells. Blood 2012; 119: 4430–4440. 22438251 10.1182/blood-2011-11-392324 PMC3362361

[10] Kanamori M , Nakatsukasa H , Okada M *et al* Induced Regulatory T cells: their development, stability, and applications. Trends Immunol 2016; 37: 803–811. 27623114 10.1016/j.it.2016.08.012

[11] Jeffery HC , Jeffery LE , Lutz P *et al* Low‐dose interleukin‐2 promotes STAT‐5 phosphorylation, Treg survival and CTLA‐4‐dependent function in autoimmune liver diseases. Clin Exp Immunol 2017; 188: 394–411. 28176332 10.1111/cei.12940 PMC5422719

[12] Shevach EM , Thornton AM . tTregs, pTregs, and iTregs: similarities and differences. Immunol Rev 2014; 259: 88–102. 24712461 10.1111/imr.12160 PMC3982187

[13] Callaghan CJ , Rouhani FJ , Negus MC *et al* Abrogation of antibody‐mediated allograft rejection by regulatory CD4 T cells with indirect allospecificity. J Immunol 2007; 178: 2221–2228. 17277127 10.4049/jimmunol.178.4.2221

[14] Bestard O , Cruzado JM , Mestre M *et al* Achieving donor‐specific hyporesponsiveness is associated with FOXP3^+^ regulatory T cell recruitment in human renal allograft infiltrates. J Immunol 2007; 179: 4901–4909. 17878390 10.4049/jimmunol.179.7.4901

[15] Hua J , Inomata T , Chen Y *et al* Pathological conversion of regulatory T cells is associated with loss of allotolerance. Sci Rep 2018; 8: 7059. 29728574 10.1038/s41598-018-25384-x PMC5935752

[16] Pilat N , Granofszky N , Wekerle T . Combining adoptive Treg transfer with bone marrow transplantation for transplantation tolerance. Curr Transplant Rep 2017; 4: 253–261. 29201599 10.1007/s40472-017-0164-7 PMC5691126

[17] Dons EM , Raimondi G , Cooper DK *et al* Non‐human primate regulatory T cells: current biology and implications for transplantation. Transplantation 2010; 90: 811–816. 20671597 10.1097/TP.0b013e3181ebf782 PMC2959150

[18] Josefowicz SZ , Lu LF , Rudensky AY . Regulatory T cells: mechanisms of differentiation and function. Annu Rev Immunol 2012; 30: 531–564. 22224781 10.1146/annurev.immunol.25.022106.141623 PMC6066374

[19] Miyara M , Sakaguchi S . Human FoxP3^+^CD4^+^ regulatory T cells: their knowns and unknowns. Immunol Cell Biol 2011; 89: 346–351. 21301480 10.1038/icb.2010.137

[20] Ohkura N , Kitagawa Y , Sakaguchi S . Development and maintenance of regulatory T cells. Immunity 2013; 38: 414–423. 23521883 10.1016/j.immuni.2013.03.002

[21] Sarris M , Andersen KG , Randow F *et al* Neuropilin‐1 expression on regulatory T cells enhances their interactions with dendritic cells during antigen recognition. Immunity 2008; 28: 402–413. 18328743 10.1016/j.immuni.2008.01.012 PMC2726439

[22] Delgoffe GM , Woo SR , Turnis ME *et al* Stability and function of regulatory T cells is maintained by a neuropilin‐1‐semaphorin‐4a axis. Nature 2013; 501: 252–256. 23913274 10.1038/nature12428 PMC3867145

[23] Szurek E , Cebula A , Wojciech L *et al* Differences in expression level of helios and neuropilin‐1 do not distinguish thymus‐derived from extrathymically‐induced CD4^+^Foxp3^+^ regulatory T cells. PLoS One 2015; 10: e0141161. 26495986 10.1371/journal.pone.0141161 PMC4619666

[24] Milpied P , Renand A , Bruneau J *et al* Neuropilin‐1 is not a marker of human Foxp3^+^ Treg. Eur J Immunol 2009; 39: 1466–1471. 19499532 10.1002/eji.200839040

[25] Miyara M , Chader D , Sage E *et al* Sialyl Lewis x (CD15s) identifies highly differentiated and most suppressive FOXP3high regulatory T cells in humans. Proc Natl Acad Sci USA 2015; 112: 7225–7230. 26015572 10.1073/pnas.1508224112 PMC4466753

[26] Miyara M , Chader D , Burlion A *et al* Combination of IL‐2, rapamycin, DNA methyltransferase and histone deacetylase inhibitors for the expansion of human regulatory T cells. Oncotarget 2017; 8: 104733–104744. 29285209 10.18632/oncotarget.10914 PMC5739596

[27] Campbell DJ . Control of regulatory T cell migration, function, and homeostasis. J Immunol 2015; 195: 2507–2513. 26342103 10.4049/jimmunol.1500801 PMC4778549

[28] Hoeppli RE , MacDonald KN , Leclair P *et al* Tailoring the homing capacity of human Tregs for directed migration to sites of Th1‐inflammation or intestinal regions. Am J Transplant 2019; 19: 62–76. 29766641 10.1111/ajt.14936

[29] Lewkowich IP , Herman NS , Schleifer KW *et al* CD4^+^CD25^+^ T cells protect against experimentally induced asthma and alter pulmonary dendritic cell phenotype and function. J Exp Med 2005; 202: 1549–1561. 16314437 10.1084/jem.20051506 PMC2213331

[30] Pesenacker AM , Bending D , Ursu S *et al* CD161 defines the subset of FoxP3^+^ T cells capable of producing proinflammatory cytokines. Blood 2013; 121: 2647–2658. 23355538 10.1182/blood-2012-08-443473 PMC3617631

[31] Dwyer KM , Hanidziar D , Putheti P *et al* Expression of CD39 by human peripheral blood CD4^+^ CD25^+^ T cells denotes a regulatory memory phenotype. Am J Transplant 2010; 10: 2410–2420. 20977632 10.1111/j.1600-6143.2010.03291.x PMC2966025

[32] Gautron AS , Dominguez‐Villar M , de Marcken M *et al* Enhanced suppressor function of TIM‐3^+^ FoxP3^+^ regulatory T cells. Eur J Immunol 2014; 44: 2703–2711. 24838857 10.1002/eji.201344392 PMC4165702

[33] Cuadrado E , van den Biggelaar M , de Kivit S *et al* Proteomic analyses of human regulatory T cells reveal adaptations in signaling pathways that protect cellular identity. Immunity 2018; 48: 1046–1059.e6. 29752063 10.1016/j.immuni.2018.04.008

[34] Salvany‐Celades M , van der Zwan A , Benner M *et al* Three types of functional regulatory T cells control T cell responses at the human maternal‐fetal interface. Cell Rep 2019; 27: 2537–2547.e5. 31141680 10.1016/j.celrep.2019.04.109

[35] Hori S , Nomura T , Sakaguchi S . Control of regulatory T cell development by the transcription factor Foxp3. Science 2003; 299: 1057–1061. 28115586

[36] Wang J , Ioan‐Facsinay A , van der Voort EI *et al* Transient expression of FOXP3 in human activated nonregulatory CD4^+^ T cells. Eur J Immunol 2007; 37: 129–138. 17154262 10.1002/eji.200636435

[37] Allan SE , Crome SQ , Crellin NK *et al* Activation‐induced FOXP3 in human T effector cells does not suppress proliferation or cytokine production. Int Immunol 2007; 19: 345–354. 17329235 10.1093/intimm/dxm014

[38] Passerini L , Rossi Mel E , Sartirana C *et al* CD4^+^ T cells from IPEX patients convert into functional and stable regulatory T cells by FOXP3 gene transfer. Sci Transl Med 2013; 5: 215ra174. 10.1126/scitranslmed.3007320 24337481

[39] Pons JA , Revilla‐Nuin B , Baroja‐Mazo A *et al* FoxP3 in peripheral blood is associated with operational tolerance in liver transplant patients during immunosuppression withdrawal. Transplantation 2008; 86: 1370–1378. 19034005 10.1097/TP.0b013e318188d3e6

[40] San Segundo D , Galvan‐Espinoza LH , Rodrigo E *et al* Regulatory T‐cell number in peripheral blood at 1 year posttransplant as predictor of long‐term kidney graft survival. Transplant Direct 2019; 5: e426. 30882031 10.1097/TXD.0000000000000871 PMC6411222

[41] Mason GM , Lowe K , Melchiotti R *et al* Phenotypic complexity of the human regulatory T cell compartment revealed by mass cytometry. J Immunol 2015; 195: 2030–2037. 26223658 10.4049/jimmunol.1500703

[42] Lau AH , Vitalone MJ , Haas K *et al* Mass cytometry reveals a distinct immunoprofile of operational tolerance in pediatric liver transplantation. Pediatr Transplant 2016; 20: 1072–1080. 27781378 10.1111/petr.12795 PMC5404744

[43] Kunicki MA , Amaya Hernandez LC , Davis KL *et al* Identity and diversity of human peripheral Th and T regulatory cells defined by single‐cell mass cytometry. J Immunol 2018; 200: 336–346. 29180490 10.4049/jimmunol.1701025

[44] Yabu JM , Siebert JC , Maecker HT . Immune profiles to predict response to desensitization therapy in highly HLA‐sensitized kidney transplant candidates. PLoS One 2016; 11: e0153355. 27078882 10.1371/journal.pone.0153355 PMC4831845

[45] Seddiki N , Santner‐Nanan B , Martinson J *et al* Expression of interleukin (IL)‐2 and IL‐7 receptors discriminates between human regulatory and activated T cells. J Exp Med 2006; 203: 1693–1700. 16818676 10.1084/jem.20060468 PMC2118333

[46] Hill JA , Feuerer M , Tash K *et al* Foxp3 transcription‐factor‐dependent and ‐independent regulation of the regulatory T cell transcriptional signature. Immunity 2007; 27: 786–800. 18024188 10.1016/j.immuni.2007.09.010

[47] Ohkura N , Hamaguchi M , Morikawa H *et al* T cell receptor stimulation‐induced epigenetic changes and Foxp3 expression are independent and complementary events required for Treg cell development. Immunity 2012; 37: 785–799. 23123060 10.1016/j.immuni.2012.09.010

[48] Wu Y , Borde M , Heissmeyer V *et al* FOXP3 controls regulatory T cell function through cooperation with NFAT. Cell 2006; 126: 375–387. 16873067 10.1016/j.cell.2006.05.042

[49] Lee SM , Gao B , Fang D . FoxP3 maintains Treg unresponsiveness by selectively inhibiting the promoter DNA‐binding activity of AP‐1. Blood 2008; 111: 3599–3606. 18223166 10.1182/blood-2007-09-115014

[50] Ouyang W , Beckett O , Ma Q *et al* Foxo proteins cooperatively control the differentiation of Foxp3^+^ regulatory T cells. Nat Immunol 2010; 11: 618–627. 20467422 10.1038/ni.1884

[51] Morikawa H , Ohkura N , Vandenbon A *et al* Differential roles of epigenetic changes and Foxp3 expression in regulatory T cell‐specific transcriptional regulation. Proc Natl Acad Sci USA 2014; 111: 5289–5294. 24706905 10.1073/pnas.1312717110 PMC3986152

[52] Kitagawa Y , Ohkura N , Kidani Y *et al* Guidance of regulatory T cell development by Satb1‐dependent super‐enhancer establishment. Nat Immunol 2017; 18: 173–183. 27992401 10.1038/ni.3646 PMC5582804

[53] Walker MR , Kasprowicz DJ , Gersuk VH *et al* Induction of FoxP3 and acquisition of T regulatory activity by stimulated human CD4^+^CD25^‐^ T cells. J Clin Invest 2003; 112: 1437–1443. 14597769 10.1172/JCI19441 PMC228469

[54] Sugimoto N , Oida T , Hirota K *et al* Foxp3‐dependent and ‐independent molecules specific for CD25^+^CD4^+^ natural regulatory T cells revealed by DNA microarray analysis. Int Immunol 2006; 18: 1197–1209. 16772372 10.1093/intimm/dxl060

[55] Kitagawa Y , Ohkura N , Sakaguchi S . Epigenetic control of thymic Treg‐cell development. Eur J Immunol 2015; 45: 11–16. 25348287 10.1002/eji.201444577

[56] Zheng Y , Josefowicz S , Chaudhry A *et al* Role of conserved non‐coding DNA elements in the Foxp3 gene in regulatory T‐cell fate. Nature 2010; 463: 808–812. 20072126 10.1038/nature08750 PMC2884187

[57] Lal G , Zhang N , van der Touw W *et al* Epigenetic regulation of Foxp3 expression in regulatory T cells by DNA methylation. J Immunol 2009; 182: 259–273. 19109157 10.4049/jimmunol.182.1.259 PMC3731994

[58] Tao R , de Zoeten EF , Özkaynak E *et al* Deacetylase inhibition promotes the generation and function of regulatory T cells. Nat Med 2007; 13: 1299–1307. 17922010 10.1038/nm1652

[59] Itzykson R , Kosmider O , Cluzeau T *et al* Impact of TET2 mutations on response rate to azacitidine in myelodysplastic syndromes and low blast count acute myeloid leukemias. Leukemia 2011; 25: 1147–1152. 21494260 10.1038/leu.2011.71

[60] Yue X , Lio CJ , Samaniego‐Castruita D *et al* Loss of TET2 and TET3 in regulatory T cells unleashes effector function. Nat Commun 2019; 10: 2011. 31043609 10.1038/s41467-019-09541-y PMC6494907

[61] Wang L , Beier UH , Akimova T *et al* Histone/protein deacetylase inhibitor therapy for enhancement of Foxp3^+^ T‐regulatory cell function posttransplantation. Am J Transplant 2018; 18: 1596–1603. 29603600 10.1111/ajt.14749 PMC6035084

[62] Wu D , Luo Y , Guo W *et al* Lkb1 maintains Treg cell lineage identity. Nat Commun 2017; 8: 15876. 28621313 10.1038/ncomms15876 PMC5481770

[63] Huang J , Wang L , Dahiya S *et al* Histone/protein deacetylase 11 targeting promotes Foxp3+ Treg function. Sci Rep 2017; 7: 8626. 28819166 10.1038/s41598-017-09211-3 PMC5561267

[64] Dahiya S , Wang L , Beier UH *et al* HDAC10 targeting regulates Foxp3 promoter, enhances T‐regulatory (Treg) function and suppresses autoimmune colitis. J Immunol 2018; 200: 54.11.

[65] Wang L , Liu Y , Han R *et al* FOXP3^+^ regulatory T cell development and function require histone/protein deacetylase 3. J Clin Invest 2015; 125: 1111–1123. 25642770 10.1172/JCI77088 PMC4362235

[66] Mathew JM , J HV, LeFever A *et al* A Phase I clinical trial with *ex vivo* expanded recipient regulatory t cells in living donor kidney transplants. Sci Rep 2018; 8: 7428. 29743501 10.1038/s41598-018-25574-7 PMC5943280

[67] Tang Q , Vincenti F . Transplant trials with Tregs: perils and promises. J Clin Invest 2017; 127: 2505–2512. 28665300 10.1172/JCI90598 PMC5490750

[68] Kleinsteuber A , Halleck F , Khadzhynov D *et al* Impact of pre‐existing comorbidities on long‐term outcomes in kidney transplant recipients. Transplant Proc 2018; 50: 3232–3241. 30577191 10.1016/j.transproceed.2018.08.028

[69] Chandran S , Tang Q , Sarwal M *et al* Polyclonal regulatory T cell therapy for control of inflammation in kidney transplants. Am J Transplant 2017; 17: 2945–2954. 28675676 10.1111/ajt.14415 PMC5662482

[70] Todo S , Yamashita K , Goto R *et al* A pilot study of operational tolerance with a regulatory T‐cell‐based cell therapy in living donor liver transplantation. Hepatology 2016; 64: 632–643. 26773713 10.1002/hep.28459

[71] Geissler EK , The ONE . Study compares cell therapy products in organ transplantation: introduction to a review series on suppressive monocyte‐derived cells. Transplant Res 2012; 1: 11. 23369457 10.1186/2047-1440-1-11 PMC3561076

[72] Shaked A , DesMarais MR , Kopetskie H *et al* Outcomes of immunosuppression minimization and withdrawal early after liver transplantation. Am J Transplant 2019; 19: 1397–1409. 30506630 10.1111/ajt.15205 PMC6482056

[73] Bohne F , Martinez‐Llordella M , Lozano JJ *et al* Intra‐graft expression of genes involved in iron homeostasis predicts the development of operational tolerance in human liver transplantation. J Clin Invest 2012; 122: 368–382. 22156196 10.1172/JCI59411 PMC3248302

[74] Feng S , Ekong UD , Lobritto SJ *et al* Complete immunosuppression withdrawal and subsequent allograft function among pediatric recipients of parental living donor liver transplants. JAMA 2012; 307: 283–293. 22253395 10.1001/jama.2011.2014

[75] Riquelme P , Haarer J , Kammler A *et al* TIGIT^+^ iTregs elicited by human regulatory macrophages control T cell immunity. Nat Commun 2018; 9: 2858. 30030423 10.1038/s41467-018-05167-8 PMC6054648

[76] He W , Chen L , Zheng L *et al* Prolonged survival effects induced by immature dendritic cells and regulatory T cells in a rat liver transplantation model. Mol Immunol 2016; 79: 92–97. 27764710 10.1016/j.molimm.2016.10.004

[77] Bashuda H , Kimikawa M , Seino K *et al* Renal allograft rejection is prevented by adoptive transfer of anergic T cells in nonhuman primates. J Clin Invest 2005; 115: 1896–1902. 15951837 10.1172/JCI23743 PMC1143588

[78] Lee K , Nguyen V , Lee KM *et al* Attenuation of donor‐reactive T cells allows effective control of allograft rejection using regulatory T cell therapy. Am J Transplant 2014; 14: 27–38. 24354870 10.1111/ajt.12509 PMC5262439

[79] MacDonald KG , Hoeppli RE , Huang Q *et al* Alloantigen‐specific regulatory T cells generated with a chimeric antigen receptor. J Clin Invest 2016; 126: 1413–1424. 26999600 10.1172/JCI82771 PMC4811124

[80] Sanchez‐Fueyo A , Sandner S , Habicht A *et al* Specificity of CD4^+^CD25^+^ regulatory T cell function in alloimmunity. J Immunol 2006; 176: 329–334. 16365425 10.4049/jimmunol.176.1.329 PMC2841032

[81] Putnam AL , Safinia N , Medvec A *et al* Clinical grade manufacturing of human alloantigen‐reactive regulatory T cells for use in transplantation. Am J Transplant 2013; 13: 3010–3020. 24102808 10.1111/ajt.12433 PMC4161737

[82] Roufosse C , Simmonds N , Clahsen‐van Groningen M *et al* A 2018 reference guide to the Banff classification of renal allograft pathology. Transplantation 2018; 102: 1795–1814. 30028786 10.1097/TP.0000000000002366 PMC7597974

[83] Bluestone JA , Buckner JH , Fitch M *et al* Type 1 diabetes immunotherapy using polyclonal regulatory T cells. Sci Transl Med 2015; 7: 315ra189. 10.1126/scitranslmed.aad4134 PMC4729454 26606968

[84] ONE‐Study . Final Report Summary ‐ THE ONE STUDY (A Unified Approach to Evaluating Cellular Immunotherapy in Solid Organ Transplantation). A Unified Approach to Evaluating Cellular Immunotherapy in Solid Organ Transplantation. CORDIS Database: European Commission 2018.

[85] Sawitzki BSS , Streitz M , Jürchott K , Hutchinson J , Geissler E , Investigators P . One study: a consortium model of clinical trials optimized for comparing new immune therapies via centralized immune monitoring. American transplant congress. Am J Transplant 2019; 19(S3):340.

[86] Issa F . The TWO Study: A Phase II trial of regulatory T cells in renal transplantation ‐ can we achieve sirolimus monotherapy?: UK Research and Innovation; 2019; Study Technical Summary. Available from https://gtr.ukri.org/projects?ref=MR%252FN027930%252F1

[87] Ma L , Zhang H , Hu K *et al* The imbalance between Tregs, Th17 cells and inflammatory cytokines among renal transplant recipients. BMC Immunol 2015; 16: 56. 26400627 10.1186/s12865-015-0118-8 PMC4581081

[88] Yapici U , Kers J , Bemelman FJ *et al* Interleukin‐17 positive cells accumulate in renal allografts during acute rejection and are independent predictors of worse graft outcome. Transpl Int 2011; 24: 1008–1017. 21752104 10.1111/j.1432-2277.2011.01302.x

[89] Itoh S , Kimura N , Axtell RC *et al* Interleukin‐17 accelerates allograft rejection by suppressing regulatory T cell expansion. Circulation 2011; 124: S187–S196. 21911812 10.1161/CIRCULATIONAHA.110.014852

[90] Xie XJ , Ye YF , Zhou L *et al* Th17 promotes acute rejection following liver transplantation in rats. J Zhejiang Univ Sci B 2010; 11: 819–827. 21043049 10.1631/jzus.B1000030 PMC2970890

[91] Koenen HJ , Smeets RL , Vink PM *et al* Human CD25highFoxp3pos regulatory T cells differentiate into IL‐17‐producing cells. Blood 2008; 112: 2340–2352. 18617638 10.1182/blood-2008-01-133967

[92] Bettelli E , Carrier Y , Gao W *et al* Reciprocal developmental pathways for the generation of pathogenic effector TH17 and regulatory T cells. Nature 2006; 441: 235–238. 16648838 10.1038/nature04753

[93] Baecher‐Allan C , Viglietta V , Hafler DA . Inhibition of human CD4^+^CD25^+high^ regulatory T cell function. J Immunol (Baltimore, Md 1950) 2002; 169: 6210–6217. 10.4049/jimmunol.169.11.6210 12444126

[94] Sakaguchi S , Sakaguchi N , Asano M *et al* Immunologic self‐tolerance maintained by activated T cells expressing IL‐2 receptor α‐chains (CD25). Breakdown of a single mechanism of self‐tolerance causes various autoimmune diseases. J Immunol (Baltimore, Md 1950) 1995; 155: 1151–1164. 7636184

[95] Thornton AM , Shevach EM . Suppressor effector function of CD4^+^CD25^+^ immunoregulatory T cells is antigen nonspecific. J Immunol 2000; 164: 183–190. 10605010 10.4049/jimmunol.164.1.183

[96] Karim M , Feng G , Wood KJ *et al* CD25^+^CD4^+^ regulatory T cells generated by exposure to a model protein antigen prevent allograft rejection: antigen‐specific reactivation *in vivo* is critical for bystander regulation. Blood 2005; 105: 4871–4877. 15713793 10.1182/blood-2004-10-3888

[97] Furukawa A , Wisel SA , Tang Q . Impact of immune‐modulatory drugs on regulatory T cell. Transplantation 2016; 100: 2288–2300. 27490409 10.1097/TP.0000000000001379 PMC5077666

[98] Kim YC , Zhang AH , Su Y *et al* Engineered antigen‐specific human regulatory T cells: immunosuppression of FVIII‐specific T‐ and B‐cell responses. Blood 2015; 125: 1107–1115. 25498909 10.1182/blood-2014-04-566786 PMC4326771

